# Modeling the health impact of legislation to limit the salt content of bread in Portugal: A macro simulation study

**DOI:** 10.3389/fpubh.2022.876827

**Published:** 2022-09-13

**Authors:** Francisco Goiana-da-Silva, David Cruz-e-Silva, Ana Rito, Carla Lopes, Magdalena Muc, Ara Darzi, Fernando Araújo, Marisa Miraldo, Alexandre Morais Nunes, Luke N. Allen

**Affiliations:** ^1^Centre for Health Policy, Institute of Global Health Innovation, Imperial College London, London, United Kingdom; ^2^Faculdade de Ciências da Saúde, Universidade da Beira Interior, Covilhã, Portugal; ^3^Center for Innovation, Technology and Policy Research, IN+, Instituto Superior Técnico, Universidade de Lisboa, Lisbon, Portugal; ^4^National Institute of Health, Porto, Portugal; ^5^Department of Public Health and Forensic Sciences, and Medical Education, University of Porto Medical School, Porto, Portugal; ^6^Epidemiology Research Unit, Institute of Public Health, University of Porto, Porto, Portugal; ^7^Appetite and Obesity Research Group, Department of Psychological Sciences, University of Liverpool, Liverpool, United Kingdom; ^8^Department of Surgery and Cancer, Faculty of Medicine, Imperial College London, London, United Kingdom; ^9^Centro Hospitalar Universitário São João, Faculty of Medicine, Porto University, Porto, Portugal; ^10^Department of Management, Centre for Health Economics and Policy Innovation, Imperial College Business School, London, United Kingdom; ^11^Centro de Administração e Políticas Públicas, Instituto Superior de Ciências Sociais e Políticas, Universidade de Lisboa, Lisboa, Portugal; ^12^London School of Hygiene and Tropical Medicine, London, United Kingdom

**Keywords:** public health, salt, policy, NCD and risk factors, nutrition

## Abstract

**Background:**

Excessive salt consumption—associated with a range of adverse health outcomes—is very high in Portugal, and bread is the second largest source. Current Portuguese legislation sets a maximum limit of 1.4 g salt per 100 g bread, but imported and traditional breads are exempted. In 2017 the Ministry of Health proposed reducing the salt threshold to 1.0/100 g by 2022, however the legislation was vetoed by the European Commission on free-trade grounds.

**Aims:**

To estimate the health impact of subjecting imported and traditional breads to the current 1.4 g threshold, and to model the potential health impact of implementing the proposed 1.0 g threshold.

**Methods:**

We gathered bread sales, salt consumption, and epidemiological data from robust publicly available data sources. We used the open source WHO PRIME modeling tool to estimate the number of salt-related deaths that would have been averted in 2016 (the latest year for which all data were available) from; (1) Extending the 1.4 g threshold to all types of bread, and (2) Applying the 1.0 g threshold to all bread sold in Portugal. We used Monte Carlo simulations to generate confidence intervals.

**Results:**

Applying the current 1.4 g threshold to imported and traditional bread would have averted 107 deaths in 2016 (95% CI: 43–172). Lowering the current threshold from 1.4 to 1.0 g and applying it to all bread products would reduce daily salt consumption by 3.6 tons per day, saving an estimated 286 lives a year (95% CI: 123–454).

**Conclusions:**

Salt is an important risk factor in Portugal and bread is a major source. Lowering maximum permissible levels and removing exemptions would save lives. The European Commission should revisit its decision on the basis of this new evidence.

## Introduction

Cardiovascular diseases are the most common cause of death globally and constitute an important public health challenge (1). In Portugal, 29% of deaths are due to CVDs (2) and the prevalence of hypertension is 42.2% (3). As shown by the Global Burden of Disease study, poor diet is the risk factor that contributes most to the loss of healthy life years among the Portuguese population (4).

The average daily intake of salt per capita among the Portuguese is 7.4 g (5, 6), which is well above the WHO's recommended maximum (<5 g/day) (7). Portugal ranks the highest in Western Europe for salt intake, with excessive intake reported in 63.2% of women and 88.9% of men (5, 6). The problem affects also younger groups with over half of all children and adolescents exceeding daily recommendations (8–10). The World Health Organization (WHO) specifically calls for interventions to reduce salt intake as one of the most cost-effective measures to improve health (7).

Data from the latest National Food, Nutrition and Physical Activity Survey (5, 6) shows that after salt added to food (11), bread is the second largest source of salt in the Portuguese diet, constituting 18.2% of daily salt intake in Portugal. The same survey showed that the mean daily consumption of bread was 100.3 g/person/day, which is higher than the consumption of cereals, cereal products and tubers (5, 6). Another study also highlighted the high salt content of school meals which commonly, in Portugal, include an added piece of bread. The study showed that not only this bread serving portion was double the recommended values (45 g instead of 25 g) but was the major contributor of salt when compared with the main dish and soup, adding a mean value of 0.48 g of salt/serving to the school meal (11). Controlling salt consumption in bread plays a central part in Portugal's salt reduction strategy. Many other countries have also considered using the reduction of salt in bread, despite the hedonic (e.g., taste) and technical (e.g., dough handling) challenges (12–14).

On 12th of August 2009 Portugal introduced legislation that limited the salt content of bread to 1.4 g salt per 100 g bread (15). However, the legislation did not cover imported or “traditional” bread, defined as (16):

- Bread products containing added meat preparations and sausage-meat;
- Regionally produced bread classified as traditional and with protected name, which has characteristics easily recognizable by consumers;
- Special bread having very specific characteristics, for which the use of other ingredients is allowed (as specified in point 5 n° 7° of paragraph n° 425/98) and standardization of a general salt limit is not suitable;
- Products which are similar and/or related to bread.

According to Nielsen data, ~90% of bread consumed in Portugal is unpackaged bread from bakeries (17). The remaining 10% is packaged bread—of which just over half is imported (6% of all bread). The National Association of Bread Producers (AIPAN) estimate that 45% of all bread sold in Portugal is “traditional” (18).

In 2017 the Portuguese Ministry of Health introduced non-binding self-regulation agreements with bread producers and distributors to meet a lower salt threshold. These agreements established that bread should not have salt content exceeding 1.0 g per 100 g of bread. On the 13th of July 2018, the Ministry of Health proposed a new bill to enshrine lower maximum levels in law, applying to all forms of bread, including imported products. The proposed bill was approved at the Government Secretaries' of State meeting and, therefore, notified to the European Commission by the Portuguese authorities (19). The proposed bill aimed to gradually reduce the threshold from 1.4/100 g to:

- 1.3/100 g by the 1st of January 2019,
- 1.2/100 g by the 1st of January 2020,
- 1.1/100 g by the 1st of January 2021, and
- 1.0/100 g by the 1st of January 2022.

The European Commission rejected the proposed legislation on the basis that it restricted the salt content of imported bread and, consequently, restricted free-trade between member states. In response to this decision a preliminary impact assessment on mortality was developed by the WHO and shared with the European Commission. This draft evidence was not considered sufficient to overturn the decision, and the internal market rationale prevailed over public health protection and promotion. This is despite the fact that the European Commission has adopted *The Strategy for Europe on Nutrition, Overweight and Obesity Related Health Issues* and launched the EU Salt Reduction Framework which recognized salt reformulation as a principal factor in achieving a reduction in salt intake among the member states. The same framework recognizes bread as one of the main sources of salt intake and an important target for intervention.

### Objectives

In this study we aimed to conduct a formal assessment of the potential health gains of the proposed bill to lower the threshold from 1.4 g to 1.0 g salt/100 g bread, and to apply these limits to traditional and imported products.

## Methods

### Data sources

#### Bread consumption

**Figure 1:**
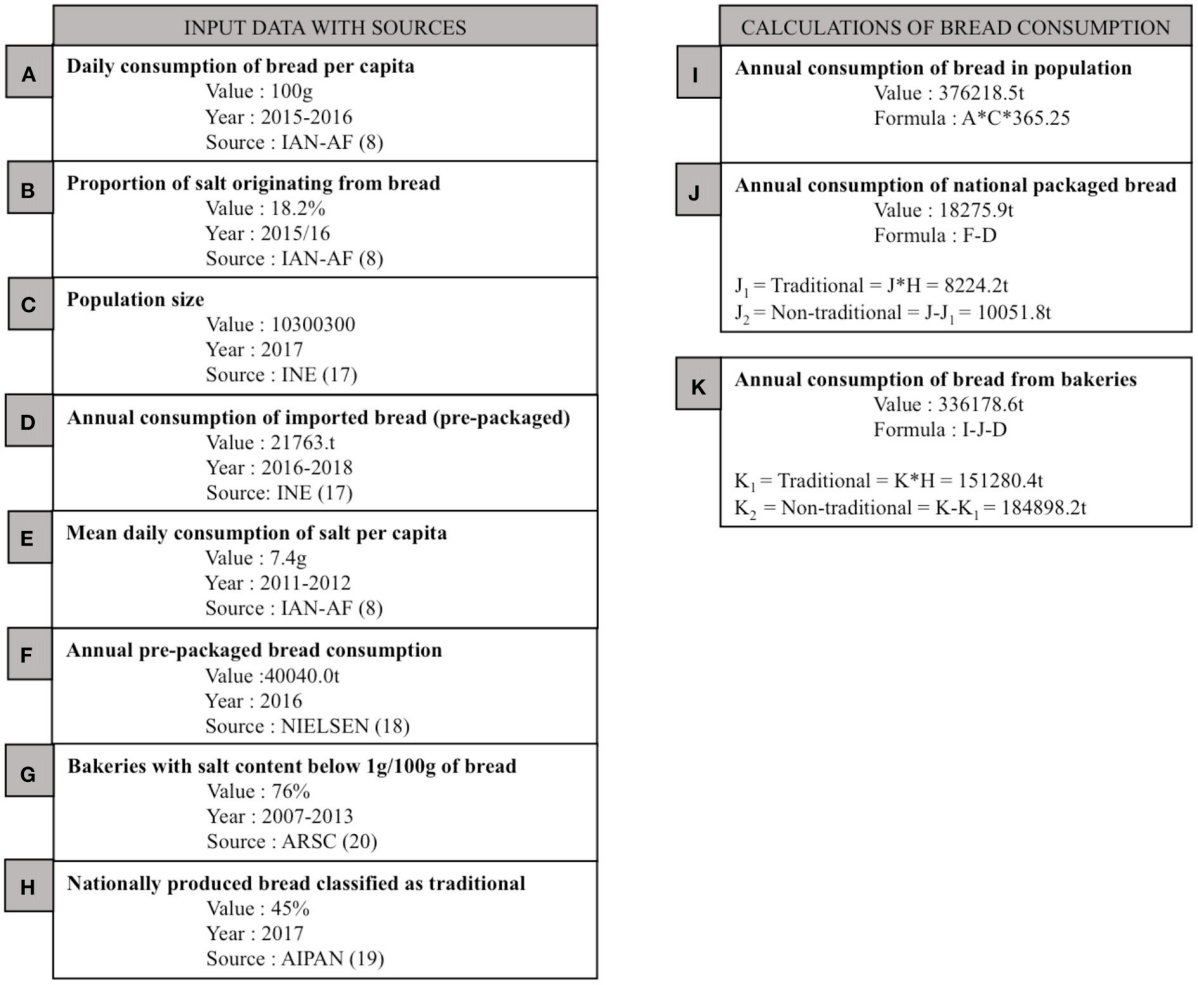
Data used in this study.

Information about the daily bread consumption in Portugal was obtained from the latest *National Food, Nutrition and Physical Activity Survey*, which estimated the daily intake at 100.3 g per capita (8). We multiplied this by the current population size (10,300,000 people) (20) and multiplied the product by 365.25 to obtain the annual intake of bread in Portugal. Figure 1 illustrates all data sources used, formulas and final estimated values.

In Portugal bread can be sold loose (in bakeries) or pre-packaged—which includes all imported bread. Data from Nielsen, a global information and measurement company, which supplements sales data with in-store observations, was used to obtain pre-packaged bread sales. Data provided by Nielsen (17) showed that in 2016 40,040 tons of pre-packaged bread was sold in Portugal. According to the National Institute of Statistics (20), 21,764 tons of pre-packaged bread is imported. These two data sources allowed us to calculate the amount of nationally produced pre-packaged bread. Finally, the difference between the total bread sold in the country and packaged bread was used to calculate the amount of bread sold loose in bakeries.

As bread classified as traditional is exempt from current legislation, we needed to know what proportion of bread falls under this category. AIPAN (National Association of Bread Producers) declared that 45% of all bread produced in Portugal is classified as “traditional bread” (18).

Using the amount of imported and traditional bread allowed us to calculate the amount of bread which is currently exempt from legislation limiting salt contents.

### Salt in bread

Next, we proceeded to estimate the salt intake from bread available on the Portuguese market. To calculate the amount of salt coming from all bread sold in Portugal, we used data from the National Food, Nutrition, and Physical Activity Survey of the Portuguese General Population (2015–2016). This data shows that 18.2% of salt intake in the population comes from bread (5, 6). According to the same survey, average daily consumption of salt per capita is 7.4 g. We multiplied the daily salt intake per capita by the population size to estimate the total salt intake per day.

### Simulations

We estimated the total reduction in salt intake that would be observed if all bread sold in Portugal (including imported, domestic, and traditional bread) complied with legislation establishing salt limits, using two simulations:

### Counterfactual scenario 1

We calculated the total reduction in salt consumption if all bread products (including imported and traditional) were to comply with the pre-existing legislation of 1.4 g of salt per 100 g of final product.

### Counterfactual scenario 2

We calculated the total reduction in salt consumption that would be observed if the maximum salt level of <1.0 g salt/100 g bread was applied to all bread sold in Portugal (imported, domestic, and traditional).

#### Modeling health gains

We used the open-source PRIME NCD mortality modeling tool developed by researchers at Oxford University, and endorsed by the WHO Regional Office for Europe, which is described in detail elsewhere (21, 22) PRIME calculates how many deaths would have occurred in the baseline year if the distribution of risk factors—in our case daily salt consumption—had been different, based on relative risk figures from peer-reviewed meta-analyses. Causes included cerebrovascular disease, ischaemic heart disease, heart failure, aortic aneurism, pulmonary emboli, rheumatic heart disease and hypertensive disease. We used Monte Carlo analysis to generate confidence intervals; effectively compiling uncertainty around a deterministic result. We used the established daily salt consumption value of 7.4 g per person per day as the baseline, taken from the National Food, Nutrition, and Physical Activity Survey of the Portuguese General Population (5, 6). Appendix 1 presents the demographic and epidemiological data used, taken from the National Institute for Statistics (INE) and the WHO Global Health Estimates (23).

### Sensitivity analyses

In order to account for potential biases on our calculations we ran several sensitivity analyses.

#### Sensitivity analysis 1: Proportion of bakeries with 1 g of salt per 100 g of final product

**Sensitivity analysis 1a:** According to the bakeries' national association, 76% of bakeries already use the 1.0 g threshold. This is built into our main model. However, to hedge against the possibility that all bakeries now comply with the 1.0 g threshold we ran a sensitivity analysis where compliance with the voluntary target was 100%.

**Sensitivity analysis 1b:** A more likely situation is that 76% is an overestimate. To hedge against this we performed a sensitivity analysis where compliance was 50%.

#### Sensitivity analysis 2: Higher salt intake baseline

The PHYSA study from 2011 to 2012 estimated average daily consumption of salt to be 10.7 g per capita using urinary sodium—a very reliable method (3). We re-ran the analyses using this higher baseline salt consumption (10.7 g/day/capita rather than 7.4/day/capita):

**Sensitivity analysis 2a:** Baseline value of 10.7 g/day/capita, with all products meeting the 1.4/100 g threshold.

**Sensitivity analysis 2b:** Baseline value of 10.7 g/day/capita, with all products meeting the 1.0/100 g threshold.

#### Sensitivity analysis 3: Proportion of salt coming from bread

We performed two sets of sensitivity analyses where we changed the proportion of salt consumption that comes from bread by ±1 percentage point. We did both analyses for each of the two counterfactual scenarios i.e., CF1 - with all products meeting the 1.4/100 g threshold (sensitivity analyses 3a CF1 and 3b CF1); and CF2 - with all products meeting the 1.0/100 g threshold (sensitivity analysis 3a CF2 and 3b CF2).

**Sensitivity analysis 3a CF1:** Proportion of salt originating from bread 1% higher with all products meeting the 1.4/100 g threshold.

**Sensitivity analysis 3b CF1:** Proportion of salt originating from bread 1% lower with all products meeting the 1.4/100 g threshold.

**Sensitivity analysis 3a CF2:** Proportion of salt originating from bread 1% higher with all products meeting the 1.0/100 g threshold.

**Sensitivity analysis 3b CF2:** Proportion of salt originating from bread 1% lower with all products meeting the 1.0/100 g threshold.

#### Sensitivity analysis 4: Lower baseline of daily salt intake

Our final set of sensitivity analyses used a 1% lower baseline salt intake, applied to the 1.4 and 1.0 g thresholds, respectively.

**Sensitivity analysis 4a:** Baseline of daily salt intake 1% lower, with all products meeting the 1.4/100 g threshold.

**Sensitivity analysis 4b:** Baseline of daily salt intake 1% lower, with all products meeting the 1.0/100 g threshold.

## Results

The Portuguese population consumes ~376, 218 tons of bread annually. The amount of nationally produced pre-packaged bread has been estimated to account for 4.86% of all bread consumed (18,276 tons/year) and 89.36% of all bread is baked fresh at national bakeries (336,179 tons/year).

We estimated that 48.18% (181,268 tons/year) of all sold bread is currently exempt from legislation defining the maximum value of salt in bread. Of this excluded bread, exactly 88% is exempt because it is traditional and the remaining 12% is exempt because it is imported. See Figure 1 for other results.

If all bread complied with the current law (1.4 g per 100 g bread salt maximum), we estimate that 1.4 fewer tons of salt would be consumed daily in Portugal. This translates to a decrease of 0.13 g of salt per day per person. Using PRIME, we estimate that an 0.13 g/day/person reduction in salt consumption would avert 107 deaths (95% CI: 43–172) per year, holding all other variables constant (Table 1). Whilst men consume more salt than women, there are many more older women in Portugal, such that 46 averted deaths were among men (95% CI: 19–74) and 61 were among women (95% CI: 25–99). If we focus exclusively on deaths averted in those aged under 75 years, we find that 16 deaths were averted among males (95% CI: 6–26) vs. 9 among women (3–14).

**Table 1:** Deaths averted by applying the established 1.4 g salt/100 g bread threshold to all bread products sold in Portugal.

	**Description**	**Baseline salt consumption (g/day/capita)**	**Absolute salt reduction (g/day/capita)**	**Counterfactual (i.e., new) salt consumption (g/day/capita)**	**Deaths averted annually (95% CI)**
Counterfactual scenario 1	All products <1.4 g salt/100 g bread	7.40	0.13	7.27	107 (43–172)
Sensitivity analysis 1a	Proportion of bakeries with <1 g salt moves from 76 to 100%	7.40	0.18	7.22	148 (59–235)
Sensitivity analysis 1b	Proportion of bakeries with <1 g salt moves from 76/24% to 50%/50%	7.40	0.08	7.32	67 (26-109)
Sensitivity analysis 2a	Different baseline (10.7 g), all products <1.4 g salt/100 g bread	10.70	0.73	9.97	592 (243–932)
Sensitivity analysis 3a	1% more salt comes from bread, all products <1.4 salt/100 g bread	7.40	0.21	7.19	173 (71–273)
Sensitivity analysis 3b	1% less salt comes from bread, all products <1.4 salt/100 g bread	7.40	0.06	7.34	50 (20-84)
Sensitivity analysis 4a	Different baseline (1% lower), all products <1.4 salt/100 g bread	7.33	0.12	7.21	99 (40–160)

In our most conservative sensitivity analyses, where the proportion of salt coming from bread falls by 1%, we estimate that between 20 and 84 lives would be saved. In our analysis that used the PHYSA salt consumption baseline we estimated that 592 deaths would be averted (95% CI: 243–932)—this is five times higher than our main estimate.

If all bread products met the proposed threshold of 1.0 g of salt per 100 g of bread we estimate that this would decrease salt consumption by 3.6 tons/day, translating into a decrease of 0.35 g of salt per day per capita. Using PRIME, we estimate that a 0.35 g/day/person reduction in salt consumption would avert 286 deaths (95% CI: 123–454) per year, holding all other variables constant (Table 2). In terms of sex-specific outcomes, 127 averted deaths were in males (95% CI: 54–200) and 159 in women (95% CI: 69–254). Again, this reversed in the under-75s, with 44 male deaths averted (95% CI: 19–70) vs. 22 female deaths (95% CI: 10–35).

**Table 2:** Deaths averted by applying the mooted 1.0 salt/100 g bread threshold to all bread products sold in Portugal.

	**Description**	**Baseline salt consumption (g/day/capita)**	**Absolute salt reduction (g/day/capita)**	**Counterfactual (i.e., new) salt consumption (g/day/capita)**	**Deaths averted annually (95%CI)**
Counterfactual scenario 2	All products <1.0 g salt/100 g bread	7.40	0.35	7.05	286 (123–454)
Sensitivity analysis 2b	Different baseline (10.7 g),	10.70	0.95	9.75	768 (328–1,212)
Sensitivity analysis 3a	1% more salt comes from bread	7.40	0.42	6.98	343 (139–542)
Sensitivity analysis 3b	1% less salt comes from bread	7.40	0.27	7.13	221 (98–351)
Sensitivity analysis 4b	Different baseline (1% lower)	7.33	0.33	6.99	271 (111–432)

In our most conservative sensitivity analysis, with 1% less salt coming from bread, the number of averted deaths dropped to 221, however the confidence interval overlaps with the main estimate (95% CI: 98–351). If the actual population baseline salt consumption is 10.4 g—in line with the PHYSA study, then we estimate that 768 deaths would be averted (95% CI: 328–1,212).

## Discussion

Nearly half of all bread sold in Portugal is exempt from the current legislation. Extending the existing 1.4/100 g threshold to all bread products would reduce daily salt intake by around 2% and save over 100 lives each year. Imposing the stricter 1.0/100 g threshold would cut salt intake by around 5% and prevent over 250 deaths each year.

Our sensitivity analyses highlight the fact that the total number of averted deaths is dependent on our assumptions about baseline salt intake. However, even if the proportion of salt coming from bread was 1% lower than survey data suggest, we would still observe around 99 lives saved each year with the extension of the current 1.4 g threshold to imported and traditional products, or 271 lives saved with the lower threshold.

Despite the fact that imported bread plays a relatively small role in comparison with traditional bread, Portugal's experience with the European Commission highlights an important and ubiquitous policy issue around the trade-off between health and market competition. The High Level Group on Nutrition and Physical Activity of the European Commission recommends that Member States implement national initiatives for the reduction of salt. In particular, it encouraged Member States to focus on food categories recognized as main sources of salt intake, including bread (24). This builds on the EU framework for salt reduction that was published in 2008 with the aim of promoting salt reduction and meeting national and WHO recommendations (7). This framework supports national plans, while at the same time preserving the necessary flexibility for State Members to formulate their own strategies toward salt reduction. In their systematic review, Santos and colleagues identified 57 countries that have established salt reformulation targets, including many variations of regulated limits on salt content in bread within European countries (25).

The EC salt framework supports measurable actions and assumes food groups representing the main source of salt intake (i.e., bread) should be a priority. It was based on this premise that the Portuguese Ministry of Health reached out to the European Commission to request permission to extend the national law and include imported bread.

In general, Member States are free to adopt whatever rules on food composition they consider necessary to protect public health in their territories, as long as these rules are adopted consistently with the EU internal market law and general principles of EU law as a whole (C-174/82 *Sandoz*). The European Commission rejected the government's initial proposal on the basis that it would restrict Portuguese access to bread legally produced and sold in the other Member States. It is important to note that the original estimates supplied were draft figures. This may have played a role in influencing the Commission's decision. In its ruling, the Commission recalled case law on the regulation of salt content in bread, which established that placing limits on salt content constituted a measure with equivalent effect to a quantitative restriction, which conflicts with Article 34 TFEU (C-17/93 *Van der Veldt*; C-123/00, *Bellamy*).

A national rule that conflicts with Article 34 TFEU can be justified (and allowed to stand) if it pursues a legitimate objective of protecting public health (Article 36 TFEU; C-120/78 *Cassis*) and is proportionate to that objective. A rule is proportionate if it is appropriate to and necessary for achieving that objective, and a measure is considered unnecessary “if human life and health can be as effectively protected by measures that are less restrictive of trade within the European Union” (C-333/14 *Scotch Whisky Association*, para 41).

Member States are responsible for demonstrating that their measure is necessary for the protection of public health using the available scientific evidence (C-333/08 *Commission v France*). A consistent line of case law has established that “specific evidence” must be presented to justify trade restrictive public health rules: Member States must prove that the new law will contribute to the protection of public health (C-148/15 *Deutsche Parkinson Vereinigung*; C-456/10 *ANETT*; C-254/05 *Commission v Belgium*; C-319/05 *Commission v Germany*).

Our study provides evidence that lower maximums in bread products are likely to save lives. This aligns with modeling work from Trieu et al. who found that even small reductions in salt intake from reformulation efforts are associated with large reductions in mortality. They also used data from an Australian national nutrition survey and sales data from Nielsen (26). This work also builds on an allied review by Hyseni et al. showing that mandatory reformulation is the most effective measure for achieving population-level reduction in consumption of *trans* fats, having an impact ~3.6 times larger than labeling (27)—an alternative strategy that the CJEU has suggested would be less trade restrictive (C-17/93 *Van der Veldt*). A major advantage of reformulation over other types of nutrition intervention is that success is not dependent on personal willpower.

Given the success of the bread reformulation already achieved by the Portuguese government, the reformulation of traditional bread provides an opportunity to tackle a significant source of salt intake and save hundreds of lives annually. Registering products as “traditional” serves the purpose of preserving an important element of identity and heritage associated with traditional food. Whilst this is important, we feel that it should not come at the price of poor health and lost lives, especially considering its large representation among the consumed bakery products. Revision of the decision to protect these large group of breads from the current legislation should be a priority if the salt reduction target is to be achieved.

Given the large number of deaths that can be averted with bread reformulation, we recommend that the Portuguese Ministry of Health look to other sources such as soups and processed meats (the next largest contributors after bread and salt added to food), using the recently published WHO global sodium benchmarks (28).

Globalization and free-trade are linked to a shift toward the consumption of more processed foods and foods high in sugar, salt and fat (29). The “Health in All Policies” approach to policymaking (30) is a response to this trend, and if implemented effectively would help policymakers to recognize where trade policy might compromise health protection, and take steps to establish a better balance between these two public goods. While some might suggest that EU free movement rules favor the promotion of trade and prevent the adoption of evidence-based public health policies—thus contradicting the imperatives of Health in All Policies—EU internal market law was always framed, and has continued to develop, to accommodate the conflict between health and trade objectives. Internal market law protects the prerogative of Member States to prioritize the protection of public health objectives over trade objectives. A balance between the two objectives is struck through an examination of the evidence—a public health measure that restricts free trade is acceptable as a matter of EU law if evidence demonstrates that it secures real public health benefits and that it is more effective at securing those benefits than other less trade restrictive measures. Although including traditional bread in the current legislation would classify as such measure, we believe that these two interventions should not be exclusive but applied alongside each other to achieve an optimal public health gains and save lives of Portuguese people.

## Limitations

We were unable to obtain data for all of our inputs for the same year, however most of our data years overlapped with 2015/2016. Some of our input data are already estimations such as the fraction of bread classified as traditional by AIPAN (31) and the percentage of salt intake coming from bread (5, 6). Therefore, calculated values should be treated as estimations and not as exact values. The values of the pre-packaged bread provided by Nielsen includes all the data from retail stores in Portugal where the vast majority of packed bread is sold. However, the small proportion of pre-packaged bread sold through other channels is not included which can cause an underestimation of its sales values.

The ARSC sample used to calculate the percentage of bakeries complying with 1.0/100 g of bread limits refers to the central region where projects such as pao.come worked proactively to reduce the salt amount. We predict that on the national level the fraction of bakeries following the 1 g limit may be lower. Thus, the impact of the new legislation may be higher than the calculated in this study.

Another limitation is the lack of information about the salt content of imported and traditional bread, which frustrates estimates of the contribution of each of these groups to salt intake.

The same source was used for the daily amount of bread intake and the daily salt intake by the Portuguese population: the National Dietary Survey IAN-AF. However, salt intake levels are typically underreported by dietary surveys. This may lead to an underestimation of the number of deaths averted, and the higher baseline salt intake value from sensitivity analysis 2 may be a more accurate estimate for the impact of the two thresholds.

Changing the salt content of bread may shift consumers preferences toward other products that are high in salt. Our study does not factor-in potential substitution effects. However, while we cannot rule out this possibility, a recent meta-analysis shows that salt reduction in bread of up to 40% does not impact consumer acceptability or elasticity of demand (32). We estimate that applying the 1.4 and 1.0 g thresholds leads to respective 9.9 and 25.7% falls in the salt content of Portuguese bread, so these effects are unlikely to affect our findings.

Finally, our model only permitted the estimation of deaths averted from salt reformulation. By not including morbidity and incidence of non-fatal diseases we significantly underestimate the likely health impact of extending legislation to cover all breads and tightening the maximum threshold.

## Conclusions

Despite the proven effectiveness of the mandatory reformulation in reducing population salt intake, half of the bread in the Portuguese market (traditional and imported bread) is currently exempt from national legislation. The proposed bill setting a maximum levels of 1.0 g salt per 100 g of final product aimed to address this but was initially rejected by the European Commission—partly on the basis of the level of evidence available. Here we present robust evidence that implementing this bill and extending it to all forms of bread is likely to save hundreds of lives each year.

## Data availability statement

The original contributions presented in the study are included in the article/Supplementary material, further inquiries can be directed to the corresponding author/s.

## Author contributions

FG-d-S conceived the study and drafted the initial manuscript. LA wrote the final draft. All authors contributed to methods and data analysis, reviewed, and edited the manuscript.

## Funding

This study was funded by Imperial College London.

## Conflict of interest

Author FA was the Portuguese Secretary of State for Health until October 2018. Authors FG-d-S and DC-e-S were members of the Portuguese Secretary of State for Health office until October 2018. The remaining authors declare that the research was conducted in the absence of any commercial or financial relationships that could be construed as a potential conflict of interest.

## Publisher's note

All claims expressed in this article are solely those of the authors and do not necessarily represent those of their affiliated organizations, or those of the publisher, the editors and the reviewers. Any product that may be evaluated in this article, or claim that may be made by its manufacturer, is not guaranteed or endorsed by the publisher.
