# Supplementary material for: Modeling the health impact of legislation to limit the salt content of bread in Portugal: A macro simulation study
Source: Front Public Health. 2022 Sep 13;10:876827. doi: 10.3389/fpubh.2022.876827 (PMC9513608; doi:10.3389/fpubh.2022.876827)
Supplement: Supplementary file 1 [file Data_Sheet_1.docx]

**Appendix 1: Baseline data used in the model**

| **Population:** | |  |
| --- | --- | --- |
|  | Male | Female |
| **15-19** | 285539 | 273725 |
| **20-24** | 274167 | 267677 |
| **25-29** | 277458 | 277226 |
| **30-34** | 305934 | 321499.5 |
| **35-39** | 358175 | 388286.5 |
| **40-44** | 387083.5 | 422322 |
| **45-49** | 361957.5 | 394938 |
| **50-54** | 359277.5 | 397944 |
| **55-59** | 335611 | 373651 |
| **60-64** | 304205.5 | 348418.5 |
| **65-69** | 275790 | 324480.5 |
| **70-74** | 221160.5 | 283280.5 |
| **75-79** | 180312.5 | 250021 |
| **80-84** | 132988.5 | 211199.5 |
| **85+** | 89,119 | 190,381 |

**Baseline mortality by sex and age**

M: Male, F: Female. Note that salt is not a risk factor for many of these conditions, however the PRIME model requires data for each of these non-communicable diseases

| **:** | | | |  | |  | | |  | | |  | | | |  | | |  | | | |  | | |  | | | |  | | | |  |  | |  |  |  |  |  |  |  |  |  |  |  |  |  |
| --- | --- | --- | --- | --- | --- | --- | --- | --- | --- | --- | --- | --- | --- | --- | --- | --- | --- | --- | --- | --- | --- | --- | --- | --- | --- | --- | --- | --- | --- | --- | --- | --- | --- | --- | --- | --- | --- | --- | --- | --- | --- | --- | --- | --- | --- | --- | --- | --- | --- |
|  | I60-I69: Cerebrovascular diseases | I20-I25: Ischaemic heart diseases | C00-C14: Lip, oral cavity and pharynx | | C15: Oesophagus | | C16: Stomach | C34: Bronchus and lung | | C25: Pancreas | C18-20: Colorectum | | C50: Breast | C54.1: Endometrium | C23: Gallbladder | | C64: Kidney | I10-I15: Hypertensive disease | | E11,E14: Diabetes | C67: Bladder cancer | C22: Liver cancer | C53: Cervix cancer | J40-J44: Chronic obstructive pulmonary disease | K70, K74: Liver disease | | I50: Heart failure | I71: Aortic aneurysm | I26: Pulmonary embolism | | I05-09: Rheumatic heart disease | N18: Chronic renal failure | **Total** | | |  |  |  |  |  |  |  |  |  |  |  |  |  |  |
| M15-19 | 0 | 2 | 0 | | 0 | | 0 | 0 | | 0 | 0 | | 0 | 0 | 0 | | 1 | 0 | | 0 | 0 | 0 | 0 | 0 | 0 | | 0 | 0 | 1 | | 0 | 0 | 4 | | |  |  |  |  |  |  |  |  |  |  |  |  |  |  |
| M20-24 | 4 | 2 | 0 | | 0 | | 0 | 0 | | 0 | 0 | | 0 | 0 | 0 | | 0 | 0 | | 0 | 0 | 1 | 0 | 0 | 0 | | 0 | 1 | 0 | | 0 | 0 | 8 | | |  |  |  |  |  |  |  |  |  |  |  |  |  |  |
| M25-29 | 1 | 3 | 1 | | 0 | | 0 | 2 | | 0 | 2 | | 0 | 0 | 0 | | 0 | 0 | | 0 | 0 | 0 | 0 | 0 | 0 | | 0 | 1 | 0 | | 1 | 0 | 11 | | |  |  |  |  |  |  |  |  |  |  |  |  |  |  |
| M30-34 | 3 | 12 | 0 | | 1 | | 4 | 3 | | 1 | 5 | | 0 | 0 | 0 | | 1 | 0 | | 0 | 0 | 3 | 0 | 0 | 3 | | 0 | 0 | 1 | | 0 | 0 | 37 | | |  |  |  |  |  |  |  |  |  |  |  |  |  |  |
| M35-39 | 11 | 27 | 4 | | 1 | | 5 | 8 | | 1 | 8 | | 0 | 0 | 0 | | 1 | 1 | | 1 | 2 | 1 | 0 | 2 | 15 | | 1 | 7 | 1 | | 1 | 0 | 98 | | |  |  |  |  |  |  |  |  |  |  |  |  |  |  |
| M40-44 | 31 | 58 | 20 | | 4 | | 16 | 34 | | 10 | 12 | | 1 | 0 | 0 | | 2 | 0 | | 5 | 2 | 8 | 0 | 5 | 49 | | 4 | 7 | 7 | | 1 | 1 | 277 | | |  |  |  |  |  |  |  |  |  |  |  |  |  |  |
| M45-49 | 44 | 103 | 48 | | 23 | | 26 | 66 | | 19 | 47 | | 0 | 0 | 1 | | 4 | 3 | | 11 | 4 | 17 | 0 | 4 | 76 | | 6 | 7 | 6 | | 1 | 2 | 518 | | |  |  |  |  |  |  |  |  |  |  |  |  |  |  |
| M50-54 | 80 | 192 | 100 | | 40 | | 80 | 168 | | 35 | 69 | | 0 | 0 | 2 | | 8 | 6 | | 25 | 14 | 56 | 0 | 24 | 122 | | 17 | 10 | 8 | | 1 | 2 | 1,059 | | |  |  |  |  |  |  |  |  |  |  |  |  |  |  |
| M55-59 | 130 | 283 | 131 | | 64 | | 89 | 335 | | 68 | 123 | | 1 | 0 | 1 | | 17 | 13 | | 45 | 28 | 98 | 0 | 31 | 125 | | 15 | 15 | 20 | | 2 | 7 | 1,641 | | |  |  |  |  |  |  |  |  |  |  |  |  |  |  |
| M60-64 | 194 | 336 | 119 | | 79 | | 141 | 407 | | 91 | 186 | | 3 | 0 | 2 | | 29 | 18 | | 80 | 52 | 106 | 0 | 78 | 129 | | 41 | 28 | 16 | | 4 | 12 | 2,151 | | |  |  |  |  |  |  |  |  |  |  |  |  |  |  |
| M65-69 | 312 | 409 | 99 | | 69 | | 142 | 526 | | 111 | 247 | | 1 | 0 | 7 | | 32 | 34 | | 163 | 89 | 127 | 0 | 111 | 124 | | 68 | 27 | 19 | | 5 | 22 | 2,744 | | |  |  |  |  |  |  |  |  |  |  |  |  |  |  |
| M70-74 | 477 | 468 | 54 | | 59 | | 186 | 500 | | 141 | 283 | | 3 | 0 | 7 | | 39 | 58 | | 223 | 103 | 116 | 0 | 151 | 116 | | 114 | 44 | 32 | | 2 | 20 | 3,196 | | |  |  |  |  |  |  |  |  |  |  |  |  |  |  |
| M75-79 | 800 | 598 | 50 | | 53 | | 214 | 513 | | 152 | 381 | | 6 | 0 | 6 | | 51 | 104 | | 308 | 115 | 129 | 0 | 269 | 79 | | 206 | 40 | 41 | | 9 | 42 | 4,166 | | |  |  |  |  |  |  |  |  |  |  |  |  |  |  |
| M80-84 | 1181 | 671 | 35 | | 35 | | 219 | 317 | | 134 | 408 | | 5 | 0 | 9 | | 48 | 186 | | 439 | 150 | 103 | 0 | 421 | 42 | | 412 | 37 | 45 | | 10 | 91 | 4,998 | | |  |  |  |  |  |  |  |  |  |  |  |  |  |  |
| M85+ | 1820 | 1014 | 36 | | 26 | | 194 | 255 | | 97 | 442 | | 4 | 0 | 5 | | 42 | 415 | | 551 | 161 | 80 | 0 | 664 | 27 | | 943 | 31 | 65 | | 12 | 187 | 7,071 | | |  |  |  |  |  |  |  |  |  |  |  |  |  |  |
| **Total** | 5088 | 4178 | 697 | | 454 | | 1316 | 3134 | | 860 | 2213 | | 24 | 0 | 40 | | 275 | 838 | | 1851 | 720 | 845 | 0 | 1760 | 907 | | 1827 | 255 | 262 | | 49 | 386 | 27,979 | | |  |  |  |  |  |  |  |  |  |  |  |  |  |  |
|  |  |  |  | |  | |  |  | |  |  | |  |  |  | |  |  | |  |  |  |  |  |  | |  |  |  | |  |  |  | | |  |  |  |  |  |  |  |  |  |  |  |  |  |  |
| F15-19 | 1 | 0 | 0 | | 0 | | 0 | 0 | | 0 | 0 | | 0 | 0 | 0 | | 0 | 0 | | 0 | 0 | 0 | 0 | 0 | 0 | | 0 | 0 | 1 | | 0 | 0 | 2 | | |  |  |  |  |  |  |  |  |  |  |  |  |  |  |
| F20-24 | 0 | 1 | 0 | | 0 | | 0 | 0 | | 0 | 0 | | 0 | 0 | 0 | | 0 | 0 | | 0 | 0 | 0 | 1 | 0 | 0 | | 0 | 0 | 1 | | 0 | 0 | 3 | | |  |  |  |  |  |  |  |  |  |  |  |  |  |  |
| F25-29 | 1 | 1 | 0 | | 0 | | 0 | 0 | | 0 | 0 | | 2 | 0 | 0 | | 0 | 1 | | 1 | 0 | 1 | 2 | 1 | 0 | | 0 | 0 | 0 | | 0 | 0 | 10 | | |  |  |  |  |  |  |  |  |  |  |  |  |  |  |
| F30-34 | 4 | 3 | 0 | | 0 | | 2 | 1 | | 0 | 6 | | 7 | 1 | 0 | | 0 | 0 | | 0 | 0 | 0 | 2 | 0 | 0 | | 0 | 2 | 1 | | 0 | 1 | 30 | | |  |  |  |  |  |  |  |  |  |  |  |  |  |  |
| F35-39 | 8 | 2 | 1 | | 0 | | 7 | 2 | | 3 | 7 | | 36 | 1 | 0 | | 0 | 0 | | 2 | 0 | 1 | 5 | 0 | 1 | | 0 | 0 | 7 | | 0 | 0 | 83 | | |  |  |  |  |  |  |  |  |  |  |  |  |  |  |
| F40-44 | 19 | 17 | 0 | | 0 | | 10 | 25 | | 5 | 21 | | 71 | 0 | 1 | | 2 | 2 | | 0 | 2 | 3 | 11 | 3 | 16 | | 2 | 2 | 5 | | 0 | 0 | 217 | | |  |  |  |  |  |  |  |  |  |  |  |  |  |  |
| F45-49 | 33 | 18 | 5 | | 0 | | 23 | 29 | | 9 | 27 | | 88 | 0 | 0 | | 3 | 1 | | 9 | 1 | 5 | 18 | 4 | 21 | | 0 | 4 | 8 | | 0 | 3 | 309 | | |  |  |  |  |  |  |  |  |  |  |  |  |  |  |
| F50-54 | 30 | 35 | 9 | | 4 | | 38 | 62 | | 17 | 53 | | 121 | 3 | 0 | | 1 | 3 | | 15 | 5 | 9 | 17 | 3 | 35 | | 3 | 2 | 11 | | 1 | 1 | 478 | | |  |  |  |  |  |  |  |  |  |  |  |  |  |  |
| F55-59 | 62 | 59 | 3 | | 8 | | 47 | 89 | | 34 | 77 | | 134 | 14 | 6 | | 5 | 6 | | 19 | 1 | 17 | 17 | 11 | 37 | | 12 | 5 | 7 | | 3 | 3 | 676 | | |  |  |  |  |  |  |  |  |  |  |  |  |  |  |
| F60-64 | 102 | 78 | 14 | | 7 | | 65 | 103 | | 56 | 102 | | 149 | 17 | 4 | | 10 | 10 | | 59 | 11 | 20 | 16 | 19 | 32 | | 18 | 3 | 30 | | 3 | 4 | 932 | | |  |  |  |  |  |  |  |  |  |  |  |  |  |  |
| F65-69 | 175 | 123 | 16 | | 3 | | 72 | 119 | | 60 | 133 | | 183 | 28 | 10 | | 13 | 25 | | 94 | 10 | 34 | 20 | 29 | 23 | | 33 | 4 | 28 | | 10 | 13 | 1,258 | | |  |  |  |  |  |  |  |  |  |  |  |  |  |  |
| F70-74 | 363 | 219 | 11 | | 4 | | 85 | 126 | | 84 | 165 | | 175 | 41 | 11 | | 16 | 70 | | 192 | 30 | 55 | 21 | 42 | 28 | | 86 | 11 | 24 | | 13 | 19 | 1,891 | | |  |  |  |  |  |  |  |  |  |  |  |  |  |  |
| F75-79 | 719 | 375 | 17 | | 9 | | 129 | 124 | | 105 | 230 | | 210 | 44 | 12 | | 21 | 124 | | 329 | 19 | 49 | 18 | 83 | 35 | | 238 | 23 | 56 | | 16 | 38 | 3,023 | | |  |  |  |  |  |  |  |  |  |  |  |  |  |  |
| F80-84 | 1370 | 602 | 20 | | 13 | | 170 | 119 | | 131 | 305 | | 207 | 43 | 15 | | 29 | 268 | | 594 | 51 | 66 | 20 | 234 | 19 | | 542 | 18 | 76 | | 23 | 82 | 5,017 | | |  |  |  |  |  |  |  |  |  |  |  |  |  |  |
| F85+ | 3757 | 1655 | 56 | | 21 | | 233 | 143 | | 174 | 519 | | 391 | 34 | 14 | | 46 | 1,082 | | 1,115 | 111 | 66 | 26 | 600 | 14 | | 2,293 | 34 | 147 | | 34 | 272 | 12,837 | | |  |  |  |  |  |  |  |  |  |  |  |  |  |  |
| **Total** | 6644 | 3188 | 152 | | 69 | | 881 | 942 | | 678 | 1645 | | 1774 | 226 | 73 | | 146 | 1592 | | 2429 | 241 | 326 | 194 | 1029 | 261 | | 3227 | 108 | 402 | | 103 | 436 | 26,766 | | |  |  |  |  |  |  |  |  |  |  |  |  |  |  |
